# Magnetic Sensing through the Abdomen of the Honey bee

**DOI:** 10.1038/srep23657

**Published:** 2016-03-23

**Authors:** Chao-Hung Liang, Cheng-Long Chuang, Joe-Air Jiang, En-Cheng Yang

**Affiliations:** 1 Department of Entomology, National Taiwan University, No. 1, Sec. 4, Roosevelt Rd., Taipei 10617, Taiwan; 2 Department of Bio-Industrial Mechatronics Engineering, National Taiwan University, No. 1, Sec. 4, Roosevelt Rd., Taipei 10617, Taiwan; 3 Graduate Institute of Brain and Mind Sciences, National Taiwan University, No. 1, Sec. 1, Ren’ai Rd., Taipei 10051, Taiwan

## Abstract

Honey bees have the ability to detect the Earth’s magnetic field, and the suspected magnetoreceptors are the iron granules in the abdomens of the bees. To identify the sensing route of honey bee magnetoreception, we conducted a classical conditioning experiment in which the responses of the proboscis extension reflex (PER) were monitored. Honey bees were successfully trained to associate the magnetic stimulus with a sucrose reward after two days of training. When the neural connection of the ventral nerve cord (VNC) between the abdomen and the thorax was cut, the honey bees no longer associated the magnetic stimulus with the sucrose reward but still responded to an olfactory PER task. The neural responses elicited in response to the change of magnetic field were also recorded at the VNC. Our results suggest that the honey bee is a new model animal for the investigation of magnetite-based magnetoreception.

Honey bees are homing social insects whose workers collect food from a distance of up to 12 km away from the hive^1^. To find their way home, the honey bees memorise olfactory cues or visual landmarks around the hive^2^,^3^,^4^,^5^. They rely on directional information from the sky compass, which is provided by the sun^6^,^7^. Even when the sun is sheltered by clouds or other obstacles, the bees can still estimate the sun’s position by the spectral pattern or the polarization pattern in the sky^8^,^9^. The distance information is measured by optic flow perceived by the bee while in flight^10^. This information is integrated and then used for navigation^11^,^12^,^13^. In addition to navigating with visual cues, honey bees reportedly also detect and use geomagnetic fields for orientation^14^,^15^,^16^,^17^,^18^,^19^,^20^,^21^.

There are two models of the magnetoreception system in terrestrial animals–chemical magnetoreception^22^ and magnetite-based magnetoreception^23^. The chemical magnetoreception system is also known as the light-dependent magnetic sense and is mediated by the ultraviolet (UV)-A/blue light photoreceptor cryptochrome^24^. This model proposes that the signals are transmitted to the neural system through the light-induced product of radical pair reactions^22^,^25^. Some animals, such as migratory birds^26^,^27^ and the blowfly^28^, American cockroach^29^,^30^, monarch butterfly^31^, and fruit fly^24^, have been suggested to use the chemical magnetoreception system. The cryptochromes are flavoproteins that contain two pigment cofactors that may be excited by light to form a transient radical pair. The reaction rates of the radical pairs depend on the strength and orientation of the outer magnetic field^25^,^26^. The UV-A/blue light photoreceptor cryptochrome is necessary for light-dependent magnetosensitive responses in *Drosophila melanogaster*. These flies have been trained to respond to the magnetic field under full spectrum light (~300–700 nm), but if the UV-A/blue part of the spectrum (<420 nm) is blocked, the flies no longer respond to the magnetic field^24^. Cryptochromes that are used in the chemical magnetoreception system also occur in the honey bee brain^32^. However, to date, there is no evidence that honey bee magnetoreception uses cryptochromes. The magnetite-based magnetoreception system suggests that the animals can sense the field through the ferromagnetic crystals of magnetite Fe_3_O_4_ located in their bodies^23^. This hypothesis derives from the discovery of the mineral magnetite in magnetotactic bacteria that have been demonstrated to orient in magnetic fields^33^,^34^. Under the different directions of the fields, the distribution of these crystals would change, which can cause a switch in the ion channels on the cellular membrane^22^. Magnetotactic bacteria^33^,^34^, algae^35^, ants^36^, sockeye salmon^37^,^38^, and several migrating birds^39^,^40^,^41^,^42^,^43^,^44^ have been suggested to use the magnetite-based magnetoreception system.

Iron granule-containing cells are present in the abdomen of the honey bee and these have had been suggested to serve as a magnetoreceptor^21^,^45^,^46^,^47^,^48^,^49^,^50^. However, cryptochromes, which are important magnetoreceptors in chemical magnetoreception systems, have also been found in the honey bee brain^32^. Although no evidence exists of cryptochrome involvement in honey bee magnetoreception so far, the cytochrome remains a candidate effector of magnetoreception in the honey bee. Previous studies have mainly focused on the location^14^,^21^,^45^,^46^,^48^,^49^, formation^46^,^48^, organization and possible magnetoreception mechanism^21^,^49^ of the iron granules. These studies have all indicated that the iron granules have the potential to be magnetoreceptors, but they have not directly confirmed the association of the iron granules with magnetoreception in the honey bee^51^,^52^,^53^. Evidence of the relationship between the iron granules and neural system is also very weak^53^. Whether the honey bees can receive magnetic information through the iron granules in the abdomen is still being debated. In our research, to test the magnetoreception of honey bee, we first followed the experiment of M. Vacha^29^, who explored the unconditioned reaction of the American cockroach, which changes its body axis when the magnetic azimuth changes^29^. Then, by using classical conditioning, we trained the honey bees to associate the magnetic stimulus with the proboscis extension reflex (PER). The successfully trained bees extended their probosces when a magnetic stimulus was present. We then severed the ventral nervous cords (VNC) of the trained bees, again provided a magnetic stimulus and observed the response. After comparing the responses of the bees before and after cutting the VNC, we can infer whether the honey bee can receive magnetic information from the abdomen iron granules. However, the most effective way to confirm a receptor is to record the neural signals that are induced by the stimulus and correspond to the receptor. In all the magnetite-based magnetoreception research, the neural responses have been recorded only in birds^44^ and fish^38^. The electrophysiological evidence for the iron granules being the magnetoreceptor in the honey bee is still lacking. In this research, we also recorded the neural signals in the honey bee when an extra magnetic field was applied, providing the first report of this kind in insects or even in invertebrates.

## Results

### The effects of changes in the magnetic azimuth on bee resting behaviour

**Figure 1:**
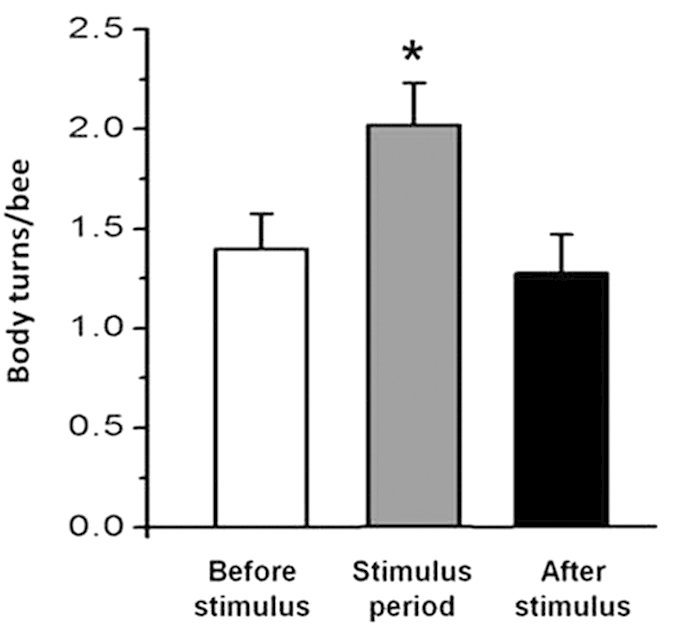
The effects of bee rest behaviour by magnetic azimuth changes. The bars indicate the number of changes in the honey bee body axis (changes greater than 10 degree) during the 90-minute periods before, during and after the change in magnetic azimuth stimulus. The experiments were conducted from 0:00 to 4:30 on every experimental day. The magnetic azimuth was changed 60° horizontally every 5 minutes during the stimulus period, and the honey bee body axis was recorded every 5 minutes during the experiment. The average body turns were 2.02 ± 0.21 (mean ± SEM) during the stimulus period (out of 18 photos), which was significantly more than before (1.40 ± 0.18) and after (1.27 ± 0.19) the stimulus period (n = 55).

To verify that honey bee behaviour is influenced by a change in the magnetic azimuth, the body turns of bees during the experimental period were recorded. The honey bees made 2.02 ± 0.21 body turns during the stimulus period (1:30 to 3:00), whereas there were only 1.40 ± 0.18 turns made before the stimulus (0:00 to 1:30) and 1.27 ± 0.19 during the after stimulus period (3:00 to 4:30). The data on body turns in response to the magnetic azimuth were analysed with a Kruskal-Wallis ANOVA, and the difference appeared to be significant (n = 55, chi-square = 8.146, df = 2, *p* = 0.017 < 0.5) (Fig. 1). This result indicates that the honey bee behaviour can be influenced by a magnetic field in a dark environment.

### PER training

**Figure 2:**
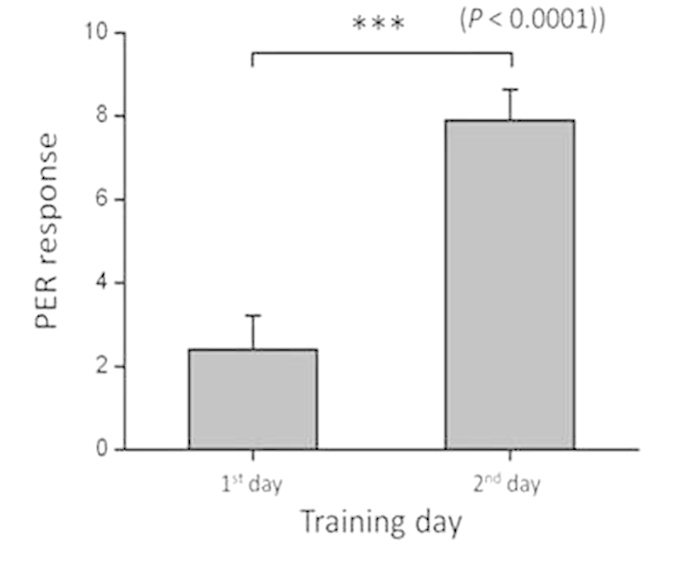
Average PER response to magnetic stimulation on the 1^st^ and 2^nd^ training days (n = 10). Each of the tested honey bees underwent 20 training trials each day. On the 1^st^ training day, the average PER responses were 2.4 ± 0.8 per 20 tests (mean ± SEM); on the 2nd day, the average PER responses increased significantly to 7.9 ± 0.7 (*t*-test, *p* < 0.0001).

In the PER experiment, each of the tested honey bees underwent 20 training trials per day. The bees seldom responded to the stimulus on the 1^st^ training day, but they responded much better on the 2^nd^ day. On the 1^st^ training day, the average conditioned stimulus (CS) PER response was 2.4 ± 0.8 times out of 20 trials (mean ± SEM); on the 2^nd^ day, the average PER response increased significantly to 7.9 ± 0.7 (mean ± SEM) (*t*-test, t value = 7.0678, *p* < 0.0001, n = 10) (Fig. 2). The success rate grew with the number of training trials completed and reached 70% at the 37^th^ training (the 17^th^ training on the 2^nd^ day). The successfully trained bees would extend their probosces during the magnetic stimulus. The data for the individuals that were dead on the 2^nd^ training day (n = 4) and had never responded to the magnetic stimulus during the two days training (n = 12) were not included in this result.

### The effects of cutting the VNC on magnetoreception

After the PER training, we cut the VNCs of the bees that had already learnt the association between the magnetic stimuli and PER (defined as the bee responding to the stimulus twice in a row). After VNC cutting, the bees no longer responded to the lateral stimulus (n = 10). As a control, the bees had also been trained to exhibit PERs to an odour stimulus, and the post-operative bees were still able to do this after VNC cutting. The bees remained 100% responsive to the odour stimulus following the first training trial after cutting the VNC, indicating that cutting the VNC at the first training trial had no influence on the odour PER. Clearly, the bees receive magnetic information through something in their abdomen, most probably the iron granules, and these signals are transmitted through the VNC.

### Neural signals in response to the field

**Figure 3:**
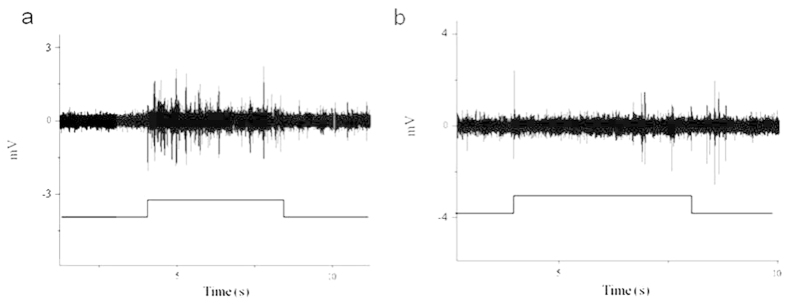
Neural responses of the magnetoreception. (**a**) Response that occurred during the stimulus. (**b**) Response that occurred after the end of the stimulus. The intensity of the field is 65 μT. The spikes indicated by the white arrow points were not the neural response but the current caused by the change of the field.

The signals that indicated response to the magnetic pulse were recorded at the VNC of the ‘neck’ of each bee. The field intensity was 65 μT, and the field direction was vertical to the geomagnetic field. The responses matched the stimulus perfectly (Fig. 3). Because of the displacement of the nervous cord, which was caused by the body fluid, it was difficult to hold cells and record the response continuously. The responding cells were easily lost after successful recording. However, two types of responses were recorded: the response that occurred during the stimulus (Fig. 3a) and the one that occurred after the stimulus (Fig. 3b). The spikes, which occurred at the same time when the stimulus has been turned on and off, were not the neural responses but the current caused by the change in the field.

## Discussion

The magnetoreception of the honey bee has been studied for decades. However, the source of the magnetic signals is still unknown. In this research, we explored whether the signals come from the abdomen. Our first experiment showed that bees can respond to changes in the magnetic azimuth in a totally dark environment. Cryptochromes, which are considered to be the magnetoreceptor in chemical magnetoreception systems, cannot function without being excited by UV-A/blue light (<420 nm)^24^. Our results show that it is unlikely that bees detect the magnetic field by using the chemical magnetoreception system but instead suggest that iron granules may participate in honey bee magnetoreception.

In previous magnetoreception studies, insects have been trained in discrimination experiments^16^,^17^,^18^,^24^. However, we preferred to use a classical conditioning experiment, which was more convenient for the application of different treatments on animals that had already been conditioned. Classical conditioning of honey bees has generally used either an odour or light stimulus to elicit a PER^54^,^55^. In our PER training experiment, we trained the bees to associate the magnetic stimulus with a sucrose reward. The bees seldom responded to the stimulus on the 1st training day, but they responded much better on the 2nd day, with the rate of success increasing with the training trials. This result revealed that bees can be trained to associate a magnetic stimulus with a sucrose reward through classical conditioning. Here, we not only confirmed honey bee magnetoreception but also provided another convenient method for conducting further studies on the magnetoreception of the honey bee.

During the PER training, we also noticed that there are several individuals that never responded to the magnetic stimulus. This finding may be a result of the method used to collect the foragers. The suspected magnetoreceptor of the honey bees, the iron granules, accumulate as bees age^47^,^48^,^49^. We did not precisely control for the age of the tested bees but captured the individuals as they flew out of the hive. The ability of magnetoreception may be influenced by the iron granules status and related to the age of the bees. Therefore the nonresponding bees may have less ability to sense the magnetic field.

Once we confirmed that the bees could sense the magnetic field, we tried to identify the source of the signal. Previous studies have mainly focused on the location^14^,^21^,^45^,^46^,^48^,^49^, formation^46^,^48^, organization and the possible magnetoreception mechanism^21^,^49^ of the iron granules. These studies all have indicated that the iron granules have the potential to be magnetoreceptors, but they have not directly confirmed the association of the iron granules with magnetoreception in the honey bee. The results of our study clearly provided evidence that the bees could no longer react to the magnetic stimulus after the connection between the iron granules and the brain was cut, thereby demonstrating that the magnetoreception signal is sensed by something in the abdomen, probably the magnetic granules, and is transmitted to the brain via the VNC.

The electrophysiological responses recorded from the VNC showed that there were two types of responses, the ON type (Fig. 3a) and the OFF type (Fig. 3b). These two types of responses have also been identified in the bobolink^44^. The neural responses of the magnetic field have been recorded only in the bobolink and rainbow trout^38^,^44^. Our data provide the first electrophysiological evidence of magnetoreception in invertebrates, and we also confirm the magnetite-based magnetoreception in honey bees. However, the connection of the iron-containing cells to the neural system remains unverified^53^. That is, how the signals transmit from the iron granules to the neural system is still an unsolved problem.

Although we have confirmed that the bees can sense a magnetic field from the abdomen, we do not have enough evidence to rule out the participation of cryptochromes in honey bee magnetoreception. The cryptochromes, which have been confirmed to be the magnetoreceptor in most other insects that can sense a magnetic field, such as the fruit fly^24^, blowfly^28^, cockroach^30^, and butterfly^31^, may also function as the magnetoreceptor in honey bee. However, the cryptochromes cannot respond to the field without being excited by light, and the honey bee needs to detect the field in their hive, for activities such as comb building^56^, in a totally dark environment. This may be the reason for the development of the magnetite-based magnetoreception in the honey bee.

## Methods and Materials

### The effects of changes in the magnetic azimuth on bee resting behaviour

Only honey bee foragers were used in this research, and this experiment was conducted in a dark room. The bees were captured in the afternoon and maintained in a plastic cup (diameter 9 cm, height 6 cm) with water and food. The cups with the bees were placed in a refrigerator (~4 °C) and the cold-anaesthetised bees were transferred individually into Petri dishes (diameter 9 cm, height 1.5 cm), which were placed on a wooden board in the coil at 20:00. On each experimental day, 18 bees were tested simultaneously, and each dish was surrounded by a strip of paper to prevent the bees from seeing each other.

The magnetic stimulus was generated by square coils. The square coils were implemented using a scheme similar to the Merritt four coil system^57^. Four sets of thin enamelled wire (Ø = 0.5 mm, ρ = 1.7 × 10^−8^ Ω·m) were wound into four coils that consisted of 26, 11, 11 and 26 turns, sequentially. The side length of each square coil was one metre, and the spacing of the neighbouring coils was consequently 37.74, 25.62 and 37.74 cm. The four coils were serially connected to a DC power supply to generate a uniform magnetic field at the central area of the coils. The coil was placed vertical to the geomagnetic field (approximately 40 μT) and generated a 65 μT field, which induced the horizontal component to rotate 60° clockwise.

The experiment took place between 0:00 and 4:30, and images of the bee behaviour were recorded every five minutes by an infrared camera, which was placed 50 cm above the wooden board. Except for the lens, the camera was wrapped in aluminium foil grounded with wire to minimise the potential influence of the magnetic field. The square coils were turned on/off to change the magnetic azimuth every 5 minutes during the stimulus period (from 1:30 to 3:00). The data of the ‘before stimulus’ (from 0:00 to 1:30) and ‘after stimulus’ (from 3:00 to 4:30) served as the control group. The images were visually checked on the computer, and we counted the body turns when the body axis slewed more than 10°.

### PER training

To examine the magnetic sensing capability of the honey bee, a magnetic field generator was built to deliver short pulses of magnetic fields to the body of the honey bee. Using ferritic stainless steel with low magnetic susceptibility (Ø = 60 mm, μ = 875 × 10^−6^ H/m, χ_m_ = 700), we constructed a semicircular magneto core with an air gap. A thin enamelled wire coil (Ø = 0.5 mm, ρ = 1.7 × 10^−8^ Ω·m) consisting of 1200 turns was wound onto the magneto core to induce a magnetic field in the air. The coil wire received an electric current whose amplitude and frequency were regulated by an external controller, which allowed the direction of the magnetic field to be modulated or the core to be demagnetised.

Only honey bee foragers were used in this research. After undergoing cold anaesthesia, the honey bees were fixed at the tip of a 1000 μL pipette with a drop of beeswax-resin mixture melted by a heated soldering iron. The compound eyes were painted over with black acrylic fabric rubber to reduce visual interference. Each honey bee was then placed at the centre of the coils overnight. The orientation of the body axis was parallel to the geomagnetic field, and the magnetic stimulus generated by the magnetic field generator was vertical to the geomagnetic field. The intensity of the stimulus was 200 μT, which is about five times the geomagnetism (approximately 40 μT), and the stimulus was 5 Hz from an alternating field.

**Figure 4:**
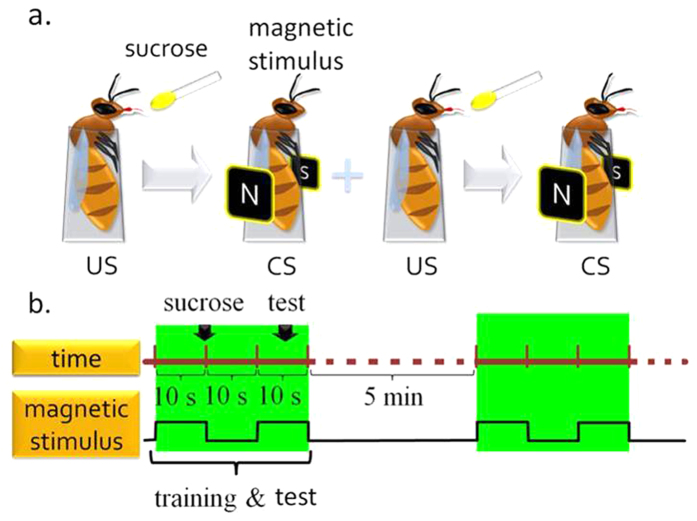
The training protocol for the proboscis extension reflex (PER) of a honey bee being conditioned by magnetic stimulus (The images of the bees were drawn by C.H.L.). (**a**) The conditioned stimulus (CS; 5 Hz of an alternating field for 10 s) was a magnetic stimulus, and the unconditioned stimulus (US) was a sucrose reward. Before training, each honey bee was first offered sucrose to verify a normal PER response to the US. Each honey bee was then conditioned by the CS, immediately followed by the US. After resting for 10 s, each honey bee was again tested by the CS to complete the training and test cycle. If a bee extended its proboscis during the test period, this suggested that it had learned the association in this trial. (**b**) The process of training and test cycles (shown in green). The interval between the two training and test cycles was 5 minutes, and we conducted 20 cycles each day.

The bees were trained to have a PER. The CS (5 Hz of alternating field for 10 s) was a magnetic stimulus, and the unconditioned stimulus (US) was a 50% sucrose reward. Before training, each honey bee was first offered sucrose to make sure that she had a normal PER response to the US. Figure 4 shows how the training and test process were conducted.

### The effects of cutting the VNC on magnetoreception

To confirm the transmission of the magnetic signals, microsurgery was used to cut the nerve connection between the iron granules in the abdomen and the brain. The successfully trained bees (those that had previously responded twice in a row to the magnetic stimulus) in PER training were used for the experiment. The surgical process was carried out as follows. (1) Bees were fixed on Styrofoam using pins that were not inserted into the honey bee’s body. (2) A small incision was made on the underside of the first abdominal sternum. The ventral nerve cord and a globe, known as the first abdominal ganglion, were visible. (3) The VNC was cut above the first abdominal ganglion to ensure that no signals from the abdomen could pass to the brain. (4) The wound was covered with petroleum jelly to reduce transpiration. After microsurgery, each bee was subjected to the magnetic stimulus again, and the response was recorded.

To eliminate the possibility that it was the surgery that resulted in the bees being unable to have a PER, the odour PER was applied as a control. The odour PER training process was conducted as follows. (1) The odour stimulus was applied for 6 seconds as the CS. (2) The sucrose reward was given immediately after the CS. (3) Then, there was a 10 second rest to allow each bee to retract its proboscis. (4) The stimulus was then reapplied to test those bees that did not follow the CS. The successfully trained bees (those that responded twice in a row to the magnetic stimulus) had their VNCs cut as described above. After surgery, the bees were again subjected to the odour stimulus, and their responses were recorded.

### Neural signals in response to the magnetic field

The magnetic field generator used in this experiment was the same as the one used to test the effects of the bee rest behaviour in response to magnetic azimuth changes. In this experiment, the glass microelectrodes were used to record the neural response. The microelectrode was made from a glass capillary tube (AF100-64-10, Sutter Instrument Co., USA), which was manufactured with a micropipette puller (Model P-87, Sutter Instrument Co., USA). The well-prepared microelectrode was sharp at the tip and tough enough to penetrate the nerve cord. A head stage was used to receive the signals from the electrode. The ‘probe+’ of the head stage was attached to a silver wire (782500, A-M Systems, Inc.), which was coated with AgCl on the surface, and then the wire was placed in the microelectrode as the recording electrode. The ‘probe-’ and the ‘probe ground’ of the head stage were tied together and attached to another silver wire as an indifferent electrode. The electrical signals could then be amplified by an AC/DC Differential Amplifier (Model 3000, A-M System, Inc., Sequim, WA, USA), transmitted to the computer and recorded by the program DataWave SciWorks (Version 7.2, DataWave Technologies Co., Loveland, CO, USA). The sampling rate was 5 kHz and we used a digital filter set as 70 Hz high pass. Finally, the response was drawn by using the program Origin (Version 7, OriginLab Co., Northampton, MA, USA).

Only foragers were used in this experiment. The bees were fixed on the pipette by using the same method used in the PER training, and all legs were removed to reduce interference. We recorded the signals from the ventral nerve cord between the thorax and the brain, which we located after removing the membrane on the ‘neck’. The indifferent electrode was placed in the mesothorax. Each bee was placed parallel to the geomagnetic field, and the magnetic field generator was placed vertical to the geomagnetic field and generated a 65 μT field, which induced the horizontal component to rotate 60° clockwise.

## Additional Information

**How to cite this article**: Liang, C.-H. *et al*. Magnetic Sensing through the Abdomen of the Honey bee. *Sci. Rep*. **6**, 23657; doi: 10.1038/srep23657 (2016).
